# Cognitive Phenotype and Psychopathology in Noonan Syndrome Spectrum Disorders through Various Ras/MAPK Pathway Associated Gene Variants

**DOI:** 10.3390/jcm11164735

**Published:** 2022-08-13

**Authors:** Ellen Wingbermühle, Renée L. Roelofs, Wouter Oomens, Jennifer Kramer, Jos M. T. Draaisma, Erika Leenders, Tjitske Kleefstra, Roy P. C. Kessels, Jos I. M. Egger

**Affiliations:** 1 Center of Excellence for Neuropsychiatry, Vincent van Gogh Institute for Psychiatry, 5803 DM Venray, The Netherlands; 2 Donders Institute for Brain, Cognition and Behaviour, Radboud University, 6525 GD Nijmegen, The Netherlands; 3 Department of Human Genetics, Radboud University Medical Center, 6525 GA Nijmegen, The Netherlands; 4 Department of Pediatrics, Amalia Children’s Hospital, Radboud University Medical Center, 6525 GA Nijmegen, The Netherlands; 5 Center of Excellence for Korsakoff and Alcohol-Related Cognitive Disorders, Vincent van Gogh Institute for Psychiatry, 5803 DN Venray, The Netherlands; 6 Department of Medical Psychology and Radboudumc Alzheimer Center, Radboud University Medical Center, 6500 HB Nijmegen, The Netherlands

**Keywords:** RASopathies, genetic disorders, cognition, cognitive phenotyping, contextual neuropsychology, psychopathology, social cognition, behavior

## Abstract

Cognitive difficulties are argued to be common in patients with Noonan syndrome spectrum disorders (NSSDs), but findings are based on studies in which patients with variants in *PTPN11* (prevalence ~50%) were overrepresented. The current study, using a structured clinical approach, describes the cognitive phenotype and psychopathology of 100 patients (aged 6 to 61 years) with nine different gene variants in the Ras/MAPK pathway underlying NSSDs (*PTPN11* *n* = 61, *PTPN11* Noonan syndrome with multiple lentigines *n* = 3, *SOS1* *n* = 14, *KRAS* *n* = 7, *LZTR1* *n* = 5, *RAF1* *n* = 4, *SHOC2* *n* = 2, *CBL* *n* = 2, *SOS2* *n* = 2). After weighted assessment and bootstrapping of the results of individual neuropsychological assessments and measures of psychopathology, cognitive performances in most variant groups were within the ranges of expectation. IQs were significantly lower in patients with variants in *PTPN11, KRAS, RAF1*, and *SHOC2*, but no specific cognitive impairments were found. The performances of younger participants (<16 years of age) did not differ from those of adults. Alexithymia and internalizing problems were more frequent in patients with variants in *PTPN11* and *SOS1*, while *PTPN11* patients also showed higher levels of externalizing problems. These results stress the need to take intelligence into account when interpreting lower cognitive performances in individual neuropsychological assessments, which is crucial for an adequate understanding and guidance of patients with NSSDs.

## 1. Introduction

Noonan syndrome spectrum disorders (NSSDs) are a group of different neurodevelopmental disorders that are caused by germline variants in multiple genes of the Ras/MAPK pathway [1]. They are estimated to occur in one out of 1000 to 2000 live births [2]. Noonan syndrome (NS; MIM# 163950) is the most prevalent and best known NSSD. It is still diagnosed primarily on clinical grounds [3]. The term NSSD refers to NS and its phenotypically related disorders; usually, neurofibromatosis is not included, as it is considered to be a clinically distinct syndrome [4]. Other NSSDs are Noonan syndrome with multiple lentigines (NSML; MIM# 151100), Noonan-like syndrome with loose anagen hair (NSLH; MIM# 607721), cardiofaciocutaneous syndrome (CFCS; MIM# 115150), and Costello syndrome (CS; MIM# 218040) [5]. Today, over 15 genes have been identified as involved in NSSD, of which the most common are *PTPN11* (50%), *SOS1* (10–13%), *RAF1* (5%), *RIT1* (5%), and *KRAS* (<5%). Table 1 lists the NSSDs with their accompanying disease genes and distinctive features. NSSDs that are caused by pathogenic variants in these genes are inherited in an autosomal dominant manner, except for *LZTR1*, which can be inherited in an autosomal recessive manner as well [3,6]. A significant percentage of cases are sporadic, due to de novo variants [5].

The phenotype of NSSDs typically comprises a variation of features, including poor growth, cardiac conditions, ectodermal anomalies, neurodevelopmental deficits, and increased neoplasm incidence [4]. However, differences in clinical manifestations are significant. See Tajan et al. (2018) [5] for an overview of the clinical features of the NSSDs.

In recent decades, studies on the cognitive phenotype of NSSDs have increased in number. Although individual variation is notably large, specific patterns may be present. Intelligence levels tend to be mildly lower, while an intellectual disability (IQ < 70) is present in less than a quarter of patients with NS or NSML [8]. Most studies that have focused on specific cognitive abilities have been performed in children and adolescents. Such studies have demonstrated impairments or weaknesses in one or more of the following domains: visual processing, motor functioning, memory, language, attention, executive functioning, and social cognition (see literature reviews [8,9]). In adults, “only” a lower speed of information processing and social cognitive impairments have been demonstrated in case-control studies [10,11]. With respect to behavior and psychopathology, children with NSSDs tend to display more symptoms of attention deficit hyperactivity disorder (ADHD) and autism spectrum disorder (ASD) than do typically developing children, which is in line with deficits found in attention and social cognition [12,13,14,15,16,17,18,19]. A higher risk of internalizing problems has been repeatedly described for both juveniles [13,14,20,21] and adults [10,22,23,24].

Today, it is largely unclear whether actual differences in intelligence, and especially in more specific cognitive abilities, exist between the NSSDs. Because pathogenic variants in the *PTPN11* gene are the most common causes of NSSDs, patients with these variants naturally dominate most study samples. Patients with NSSDs based on *SOS1* involvement display higher levels of general cognitive performance than do individuals with variants in *PTPN11* [25]. Intellectual disability is reported to be more common in CFCS, which is associated with variants in *BRAF* (~75% of the CFCS cases), *MAP2K1* and *MAP2K2* (~25%), and *KRAS* (<2–3%) [3,26,27]. These differences may reflect the differential effects of gene variants on cognitive characteristics. The aforementioned profile of somatic, cognitive, and behavioral characteristics has been described for PTPN11 variants. In the following discussion, an overview per NSSD genotype of the distinctive features and associated cognitive and behavioral characteristics is presented.

### 1.1. Cognitive and Behavioral Functioning per Pathogenic Variant

Patients with specific *PTPN11* variants that cause NSML (<1 per 100,000 live births, 90% with a variant in *PTPN11*) have characteristic pigmentary features, such as café au lait patches and lentigines. They have a high prevalence of hearing deficits (20%). Most patients display hypertrophic cardiomyopathy (HCM; 80%) [5]. Based on this more serious cardiac pathology, more cognitive disabilities and psychological challenges might be expected in this group [28]. However, an average IQ of 104 and no notable signs of psychopathology were found in a group of five children with NSML [13].

Variants in *SOS1* have been found in 10 to 13% of patients with NSSDs [3]. Heart conditions seem to be as frequent in individuals with a variation in *SOS1* as in individuals with *PTPN11* [29,30], while pulmonary valve stenosis (PVS) appears to be more frequent [29,31]. Intellectual functioning is generally within the average range, and intellectual disability is less common, compared with the general NS population [8,25,29,30,32,33]. Although the intellectual functioning of children with *SOS1* variants has been found to be significantly higher than that of children with a variant in *PTPN11* or an unknown variant [25], some studies did not find differences in IQ scores or educational needs between children and adolescents with variants in *PTPN11* and *SOS1* [19,31]. Behavioral and adaptive functioning seems to be consistent for those with *PTPN11* variants or with a clinical diagnosis of NS [13,34,35]. Interestingly, *SOS2* variants are a less frequent cause of NSSD (~4%), but both *SOS1* and *SOS2* are associated with more favorable neurodevelopment, due to changes in Ras signaling processes later in life [36,37].

*LZTR1* (~8%) [3] has quite recently been associated with NSSDs. Studies on the clinical characteristics of *LZTR1* variants are still limited, but the presentation ranges from mild to severe symptoms. HCM seems to be more prevalent in individuals with biallelic pathogenic variants, compared with individuals with heterozygous pathogenic variants [38]. The frequency of intellectual disability varies from 15.4% to 71.4% [6,37,39].

*RAF1* variants are present in approximately 5% of patients with NSSDs [3] and in 5% of patients with NSML [5]. HCM seems to be more prevalent in patients with a *RAF1* variant than in the general NSSD population [29,40]. A predisposition to hyperpigmented cutaneous lesions has been described [40]. Patients with variants in *RAF1* show intellectually impaired (55%) to normal intellectual abilities [32,40,41]. In line with intellectual impairments, similar disabilities were found in the adaptive and behavioral functioning in very small samples of patients with variants of *RAF1*, compared with patients with variants in *PTPN11* or *SOS1* [13,34].

In another 5% of the cases of NSSDs, a change in *RIT1* was found [3]. This variant is associated with a high prevalence of cardiovascular manifestations, with an overrepresentation of HCM (70–75%), as well as with more frequent lymphatic abnormalities [42,43,44]. There are indications that (cognitive) developmental impairment is present in approximately half of the patients with *RIT1* variants [45].

*KRAS* variants are found in up to 5% of patients with NSSDs [3]. *KRAS* is often associated with CFCS, although individuals tend to show high clinical variability, including phenotypes in the spectrum of Costello syndrome. Craniosynostosis, which is not a recognized feature of NS, has been reported in patients with *KRAS* variants [46]. In 2010, Pierpont et al. [34] suggested, based on the adaptive profiles of two patients with a *KRAS* variant, that they may be characterized by an intermediate phenotype between NS and CFCS regarding learning and behavior. There are indications that intellectual disabilities are more frequent and more severe in patients with *KRAS*. compared with patients with *PTPN11* or *SOS1* variants [29,32,47,48].

*BRAF* (<2% of the NSSD cases) is associated with both CFCS (80%) and NS phenotypes [3,49]. The clinical manifestations of *BRAF* are quite diverse, ranging from unimpaired cognition with only minor dysmorphic features to severe and multiple disabilities, which are more often related to CFCS. The prevalence of intellectual disability is higher in *BRAF* (90%) than in other NSSDs (6% to 28%). This can be understood from the role of *BRAF* in neurodevelopmental processes, which also explains the higher risk of epilepsy (27% versus 10% to 13% in other NSSDs). Both PVS and HCM have been associated, equally, with *BRAF* [49].

*MAP2K1* variants (<2%) have been associated predominantly with CFCS, although there are indications of milder phenotypical manifestations that resemble NS or NSML [3]. Information on neuropsychological features is sparse, but a literature overview [50] suggested that developmental delay and intellectual disability are frequent. Seizures were present in approximately half of the patients in the collected studies.

Sporadic cases of NSSDs (<1%) may be caused by changes in *SHOC2* and *PPP1CB*, which have both been linked to Noonan syndrome with loose anagen hair (NSLH). This syndrome, which is also known as Mazzanti syndrome, is characterized by hair anomalies, developmental delays, and intellectual disability related to neurological deficits, such as macrocephaly [51,52]. Furthermore, *NRAS* variants have been established in sporadic cases of individuals with NSSDs, showing typical features but no distinctive phenotype [53]. Germline variants in the *CBL* gene also constitute a very small proportion of the cases with NSSD, causing a variable phenotype that is characterized by a higher frequency of neurological features, a predisposition to juvenile myelomonocytic leukemia, and a low prevalence of cardiac conditions, reduced growth, and cryptorchidism [54]. Neuropsychological functioning in patients with variants in *NRAS* and *CBL* is largely unknown [8]. Finally, there are reports implicating variants in *RRAS* [55], *RASA2* [56], *MRAS* [57], *MAP2K2* [58], *SPRY* [56], *MAP3K8* [59], and *MYST4* [60] as rare causes of NSSD phenotypes.

Costello syndrome (CS) is caused by variants in HRAS. Cardiovascular anomalies (mostly HCM) are present in 85% of patients with CS and neurological abnormalities (both structural and functional) are common. There appears to be a rather clear neuropsychological phenotype with, in general, mild to moderate intellectual abilities, stronger nonverbal fluid reasoning in comparison to verbal reasoning and visual-spatial skills, and problems in memory and visuomotor abilities, while relative strengths have been described in facial emotion recognition and social skills. For a more detailed description of the neuropsychological profile of CS, see [61,62].

### 1.2. Study Aim and Hypotheses

Our literature overview provided a framework for the following clinical study, with the aim of evaluating the cognitive phenotypes and general psychopathology in groups of patients with an NSSD caused by different Ras/MAPK variants. Based on previous findings, IQ was expected to be low average for all groups, except for *SOS1* (higher) and the CFCS- and CS-related variations (lower). Intelligence and cognitive functioning may also be impaired in patients with variants in *SHOC2*, *PPP1CB*, and *CBL*, due to the higher prevalence of associated neurological deficits. It was anticipated that the group with *RAF1* variants might also present with cognitive problems, as they are more likely to suffer from HCM, which possibly hampers cerebral perfusion. Psychopathology, both internalizing and externalizing problems and alexithymia, was expected to be common in all groups. Younger patients (≤16 years of age) were expected to demonstrate more cognitive impairments than adults.

## 2. Materials and Methods

### 2.1. Participants

One hundred patients with NSSDs, aged 6 to 61 years, were included in this study, in the period from 2006 to mid-2021. All participants took part in larger on-going investigations; some of them were previously involved in earlier studies of the same research group [10,11,23,63,64,65,66]. Participants were recruited by the Center of Excellence for Neuropsychiatry of the Vincent van Gogh Institute for Psychiatry, Venray, The Netherlands, in collaboration with the Department of Human Genetics of the Radboud University Medical Center, Nijmegen, and the Dutch Noonan Syndrome Foundation. Inclusion criteria were (1) a confirmed NSSD with an established underlying pathogenic variant, and (2) fluency in Dutch. Genetic analyses were performed in the laboratory for DNA diagnostics of the Radboud University Medical Center. All patients underwent extensive neuropsychological assessments. Participation was voluntary and written informed consent was obtained from all participants or their legal representatives. Data collection was approved by the Institutional Review Board of the Vincent van Gogh Institute for Psychiatry, in accordance with the Declaration of Helsinki. Descriptive information of the patients is presented in Table 2. It should be noted that only patients with Noonan syndrome or Noonan-like conditions were included in the study. Patients with CFCS or CS were not actively excluded but, to date, they have not been referred to our center.

### 2.2. Materials

Data were collected by way of interviews to capture medical and developmental history and subjective information and observation on current functioning, supplemented by comprehensive neuropsychological assessments, with the results adjusted for age and education or IQ.

A broad spectrum of widely used, standardized, and sensitive neuropsychological tests was administered to assess functioning within the major cognitive domains [69]: intelligence, speed of information processing, attention, executive functioning, memory, and social cognition. Moreover, the presence or absence of alexithymia and general psychopathology were screened. The individual selection of neuropsychological tests and questionnaires was based on clinical grounds and tailored to the specific pending assessment questions, taking into account patient characteristics (e.g., age, education level. and intelligence). The tests were administered by trained assessors and testing usually took place in two days. The order of the neuropsychological tests was determined for each patient individually, depending on patient characteristics and clinical logistics. Standardized results (according to the available Dutch normative data) were used for all measurements. Given the large variability in patient characteristics, combined with the fact that individual assessments were tailored to the referral question, target domains were assessed by different sets of tasks. Supplementary Table S1 [70,71,72] provides an overview of the neuropsychological tests and questionnaires per target domain.

### 2.3. Procedure

All neuropsychological assessments were performed at the Center of Excellence for Neuropsychiatry between 2006 and 2021. All standardized results (according to adequate norm data) of the anonymized neuropsychological assessments were classified by the first two authors (E.W. and R.L.R.), in accordance with the normal distribution, as low (<−2 SD), below average (−2 to −1 SD), average (−1 to 1 SD), above average (1 to 2 SD), and high (>2 SD) for the domains of intelligence, speed of information processing, attention, executive functioning, memory, and social cognition. Note that in those tests for which no adjustment for education level or IQ was available, even though that information was relevant, the researchers themselves compared the obtained standardized scores to individual IQs, which served as a baseline reference against which other results were measured (for example, if a participant with an IQ of 90 obtained a standardized score of 70 on the BADS, this score was classified as −1 SD, whereas according to the manual, this (education-uncorrected) result would be classified as −2 SD). Classifications were thus achieved through a structured process of active interpretation and clinical judgment, in order to make valid statements about participants’ abilities and to avoid overdiagnosing and underdiagnosing [69]. Because verbal recognition measures are typically highly negatively skewed, we used established cut-off scores for “normal” and “impaired” performances for these variables [69]. Multiple sources of information from the neuropsychological assessments (self-report questionnaires, proxy-measures, anamnestic information, clinical observations, overall level of cognitive functioning) were used in order to classify results as indicative for alexithymia or psychopathology. Inconclusive classifications were resolved via consensus.

### 2.4. Data Analysis

First, separate exploratory simulation-based tests were performed for all nine variant groups and for each of 18 neuropsychological (sub)domains and variables: intelligence (FSIQ), speed of information processing (speed), sustained attention (attention), memory (verbal encoding, verbal recall, verbal recognition, visual encoding, and visual recall), executive functioning (working memory, flexibility, inhibition, and planning), social cognition (emotion recognition and mentalizing), and psychopathology (alexithymia, internalizing problems, externalizing problems, and thought disorders). To this end, the five classifications (low, below average, average, above average, high) were further converted to a dichotomous scale, reflecting either normal performance (average) or deviant performance (both low and below average, and above average and high). Therefore, a deviant performance implied that ~16% of the theoretical population had an expected standardized score of −1 SD or lower and ~16% had an expected score of +1 SD or higher, resulting in an expected proportion of ~32% altogether with deviant scores. For the binary variables of verbal recognition and psychopathology (alexithymia, internalizing problems, externalizing problems, and thought disorder), the population distribution had an expected proportion of 16%, as performances in these domains had only one direction of deviation (−1 SD or lower versus intact). 

Next, a series of exploratory simulation-based tests were performed on the FSIQs (with an expected value of 100) for all nine variant groups. Furthermore, a confirmative simulation-based inferences test was performed to compare two of the variant groups (*PTPN11* and *SOS1*) with respect to their FSIQ. Finally, the whole sample was split into two age groups (≤16 and >16 years of age) and confirmative simulation-based inferences tests were performed to compare the performances of both age groups on all 18 variables. All analyses were performed using the infer R package [73]. For a comprehensive description of the selected statistical methods see Supplementary File S1.

## 3. Results

Table 3 and Table 4 show the descriptive classifications for the cognitive domains and the psychopathology measures per variant group. The results of ten of the simulation tests were significant, as shown in Supplementary Figure S1. For these tests, the observed proportion of deviant scores was not within the range of the 95% confidence interval around the point estimate. In the *PTPN11* group, significantly more deviant scores than expected were observed regarding FSIQ, alexithymia, and both internalizing and externalizing problems. Since the psychopathology variables are dichotomous, the deviant scores in this domain reflected the presence of alexithymia and internalizing and externalizing problems in this variant group. For FSIQ, however, deviant scores reflected either more higher scores than expected, or more lower scores than expected. Therefore, Table 3 provides additional descriptive information on the direction of the deviation. In the *PTPN11* group, 50% of the FSIQ scores were lower than expected and only 5% of the scores were higher than expected, based on two-tailed testing. Furthermore, in the *PTPN11* group, significantly fewer deviant scores than expected were observed regarding attention, verbal recognition, visual recall, and working memory. In the *SOS1* group, significantly more deviant scores than expected were observed in regard to alexithymia and internalizing problems. Data for the remaining groups (*PTPN11/NSML*, *KRAS*, *LZTR1*, *RAF1*, *SHOC2*, *CBL*, and *SOS2*) did not contain sufficient variance to simulate valid distributions.

In addition to the significant results of the simulation tests, we also explored Table 3 and Table 4 for potential trends in cognitive impairments and psychopathology. Based on the two-tailed confidence intervals and using the corresponding lower and upper limits for one-tailed inspection of scores that were lower than expected (with an expected occurrence of 16%), we applied the following rules of thumb for potentially clinically relevant features: 33.3% for groups with sample sizes > 50, 66.6% for groups with sample sizes > 10, and 100% for groups with smaller samples. According to these criteria, more impairments in social cognition (emotion recognition and mentalizing) were observed in the *PTPN11* group than expected. For *PTPN11/NSML*, *SHOC2*, *CBL*, and *SOS2*, alexithymia was more present than expected. Furthermore, we observed that, in addition to *PTPN11*, externalizing problems were present in *PTPN11/NSML* but absent in all other variant groups. In addition, thought disorders were absent in all variant types except for *PTPN11/NSML* (albeit due to one classification of a thought disorder in this group of only two patients).

Because FSIQ was taken into account to classify all other cognitive performances, a series of simulation tests was performed to gain a better understanding of the aforementioned results (Supplementary Figure S2a). The *PTPN11*, *KRAS*, *RAF1*, and *SHOC2* group presented with an observed FSIQ score that was significantly lower than average (average FSIQ = 100), which may explain the lack of deviating results in these groups and the relatively good performance in attention, visual recall, and working memory in the *PTPN11* group. Furthermore, Supplementary Figure S2b depicts the direct comparison of FSIQ (simulation of difference in means) between the *PTPN11* and *SOS1* groups, showing an observed difference in mean FSIQ that was not in the range of the 95% confidence interval around the mean difference in mean FSIQ. The *SOS1* group had an observed FSIQ that was significantly higher than the observed FSIQ in the *PTPN11* group (*p*-value < 0.05).

Finally, Supplementary Figure S3 shows the comparison of the performances between younger (≤16 years of age) and older (>16 years of age) participants on each of the 18 variables. For all 18 simulation tests, all of the observed differences in performance were in the range of the 95% confidence interval around the mean difference in means. That is, there were no significant differences between the two age groups.

## 4. Discussion

In this study, intelligence, cognitive profile, and signs of psychopathology were examined in a large sample of patients with a variant in the RAS/MAPK pathway. To this end, the results of extensive neuropsychological assessments were interpreted and classified based on appropriate normative data, adjusted for age, sex, intelligence, and/or education as appropriate. Bootstrapping was used to analyze the data. This evaluation may help in understanding differences and similarities in the nature or severity of problems that patients with NSSDs encounter in daily life, such as education trajectories, work, and social functioning.

The results regarding intelligence were in agreement with expectations. The simulation tests showed that the FSIQ was lower than expected for the *PTPN11* group and, with a mean FSIQ of 85, fully in accordance with previous findings [8]. Compared with the standard of 100, the mean FSIQ of patients with pathogenic variants in *KRAS*, *RAF1*, and *SHOC2* was also evidently lower, in line with the hypotheses that were made based on previous studies [40,41,47,48,51]. Moreover, the mean FSIQ of patients with a *SOS1* variant was significantly higher than that of the *PTPN11* group, as was previously found [25] (albeit that the *p*-value for this finding was close to 0.05). Contrary to the hypothesis, the FSIQ was not significantly lower in the CBL group, which consisted of only two persons, both of whom had neurological problems (epileptic seizures and structural brain anomaly, respectively).

In contrast to our expectation, the simulation tests did not reveal any specific cognitive impairments for any of the domains/variables in any of the variant groups involved in this study, or demonstrate any differences in cognitive functioning between younger and older participants. This result differs from previous reports of empirical group studies, in some of which statistical differences in absolute performances across tasks were examined between patients and controls [9,10,11]. However, the current approach applies a clinical interpretation of these findings in relation to normative data, classifying each performance on a five-category scale in line with clinical convention. This approach, while aimed at clinical rather than statistical significance, may thus have obscured small group differences, as they may have resulted in a classification within the average range (“expected”). Moreover, the small sample size of several genetic variants results in large confidence intervals and, consequently, a high standard deviation of the sampling distribution (standard error). In turn, extremely poor performances (outliers) impact the current results to a lesser extent than in previous reports, as both extremely low performances and low performances have been classified as “lower than expected”. From a different perspective, the fact that no specific cognitive impairments were found should also be interpreted in the light of the overall low-average intellectual abilities of the patients. Because FSIQ was taken into account in the evaluation of the performances on the other cognitive tasks, the observation that cognitive capabilities are relatively limited (albeit at the same level as their intellectual abilities) became less visible. As a result, it can be assumed that in studies lacking an adjustment for intelligence, cognitive impairments in patients with NSSDs will be overestimated, as these results should always be interpreted in relation to an individual’s intellectual capacity [69]. In the same way, the lack of evidence for a difference in cognitive functioning between young and older patients may have resulted from the careful weighing of all results in this study, in which we considered individual intellectual capacities in addition to age. The cognitive performances of children are often measured against an age-adjusted norm only, while the overall level of intelligence should be considered in order to determine how low performances on specific tests may relate to general cognitive efficiency.

Notwithstanding the fact that none of the simulation tests on the cognitive data was statistically significant, an inspection of the proportion of patients per group scoring lower than expected did point toward a trend in impairment of social cognition in *PTPN11*. This observation is in line with the previous findings of studies that addressed social cognition in patients with NSSDs [10,63,65].

Regarding psychopathology, the findings largely confirmed our hypotheses, insofar as alexithymia was significantly present in two groups, *PTPN11* and *SOS1*, while it was credibly present in *PTPN11/NSML*, *SHOC2*, *CBL*, and *SOS2* as well. Less evidence could be found for a wide scope of problems in internalizing and externalizing behaviors. Internalizing behavior was again substantially present in the groups with *PTPN11* and *SOS1*, but not in any of the other groups. Externalizing behavior could only be recognized in *PTPN11* positives and, as a trend, in patients with a variant in *PTPN11/NSML*. Externalizing problems were completely absent in all other groups, a surprising finding that calls for more research, specifically focusing on differences in emotion regulation patterns in patients with NSSDs. Furthermore, participants were screened, for reasons of care and without a specific hypothesis, on the presence of thought disorders; few indications for this type of psychopathology were found (5 confirmations in 60 patients). Regarding ADHD and ASD, it should be noted that we did not record in this study, with its focus on cognition, whether patients met the criteria for these classifications, although we did investigate attention, executive functioning, and social cognition, which are the underlying cognitive domains of these clinical classifications.

The general strengths of this study include the large patient sample and the extensiveness of the assessment that all patients underwent, which included multiple cognitive and psychopathological domains. The tasks and methods used to classify the performances and to determine their clinical significance were in accordance with common practice in neuropsychology, in which individual performances are compared with age and education and/or sex adjusted norms [69].

With respect to the cognitive domains included in the assessments in this study, we consider the fact that language (production, naming, and comprehension) was not included to be a shortcoming. At the beginning of the research project (2006), only adult patients with no noticeable language disorders were referred; however, language difficulties are a known developmental problem in patients with NSSDs, a fact that argues in favor of including standard language tasks in research on the cognitive phenotype of NSSDs. Furthermore, although we used available Dutch normative data for all tests, the quality and quantity of these normative datasets vary across tests (with relatively small normative samples available for some tests), which may be considered a limitation regarding the validity of our results. However, we submit that we largely overcame this limitation by a careful interpretation of all individual results by multiple clinicians (E.W. and R.L.R.). Moreover, although the samples of some variants included in this study were small, limiting the reliability and generalizability of the results for these groups, it should be stressed that the prevalence of these variants was low. Because the primary goal of this study was to inventory cognitive and behavioral problems, detailed information regarding social backgrounds or psychiatric treatments of the patients was not included. If patients were using psychotropic drugs at the time of the assessment, any effects of such drugs on cognitive performances were included in the clinical interpretation. Future studies regarding a more in-depth analysis of psychopathology in NSSDs should, preferably, include information regarding psychiatric care and treatment history. Finally, information regarding growth hormone treatment was not systematically collected for the patients in our study. As growth hormone therapy has been associated with beneficial effects on cognition in other genetic syndromes, such as Prader–Willi syndrome [74], it would be interesting to investigate, in future studies, the relationship between cognition and growth hormone treatment in patients with NSSDs.

With regard to generalizability, it should be noted that the age range of the patients in this study was wide, and that these results are only applicable to patients with Noonan syndrome or Noonan-like conditions, but not to other NSSDs, such as CFCS and CS. Lastly, a referral bias may be present, which is hard to avoid for phenotype studies such as this study, as patients who have few or no complaints will stay out of sight of referring specialists and, consequently, will not be included in research studies. The distribution of the different variants in our study, however, largely reflects patients’ clinical epidemiology [3], even though it is unfortunate that our sample did not include patients with the relatively common defect in *RIT1* (5%).

## 5. Conclusions

Our findings demonstrate that NSSDs are characterized mainly by low-average intelligence, rather than by specific cognitive impairments. Emotion recognition, mentalizing, and alexithymia seem to be areas of concern in patients with NSSDs. Difficulties in recognizing their own and other people’s emotions may hamper emotion regulation and the building and maintaining of interpersonal relationships, resulting in behavioral problems or psychopathology. Clinicians should be aware of the psychological vulnerabilities of patients with NSSDs and pay special attention to social cognition and alexithymic tendencies. If problems in these areas interfere with daily functioning, interventions aimed at strengthening social cognition and reducing alexithymia are indicated. An example of such an intervention is social cognitive training for adults with Noonan syndrome [65].

This study illustrates the large variability in cognitive functioning, even in patients with a shared genetic mutation in the RAS/MAPK pathway. Given the large variance in performances, an individual neuropsychological assessment is indispensable in gaining insight into the cognitive and psychopathological profile of an individual with an NSSD, even when a known variant in the RAS/MAPK pathway is involved. It is recommended that individual neuropsychological assessments be performed for patients with NSSDs at key points during their development and in their adult lives, to enable tailored advice to be provided in regard to their educational support, psychosocial care, and/or psychological treatment. Neuropsychological assessments for these patients should, in general, include assessments of intelligence and all major cognitive domains, such as social cognition, supplemented with appropriate measures of psychopathology. Results should be interpreted carefully, according to common clinical neuropsychological practice, and by properly considering current and evolving knowledge on NSSDs, their genetic underpinnings, and relevant personal and contextual factors.

## Figures and Tables

**Table 1:** Noonan syndrome spectrum disorders with their associated genes and distinctive features.

NSSD	Gene	Distinctive Features
Noonan syndrome (NS)	*PTPN11*, *SOS1*, *RAF1*, *KRAS*, *NRAS*, *CBL*, *BRAF*, *SOS2*, *RIT1*, *RRAS2*, *RASA2*, *LZTR1*, *MAP2K1*, *MAP2K2*, *MRAS*	JMML (1–2%)
Noonan syndrome with multiple lentigines (NSML)	*PTPN11*, *RAF1*, *BRAF*, *MAP2K1*, *NRAS*	HCM (60–70%), multiple lentigines, sensorineural hearing deficits (20%)
Noonan syndrome with loose anagen hair (NSLH)	*SHOC2*, *PPP1CB*	Short stature (>90%), growth hormone deficiency (50%), loose anagen hair
Cardiofaciocutaneous syndrome (CFCS)	*BRAF*, *MAP2K1*, *MAP2K2*, *KRAS*, *NRAS*	Pronounced craniofacial and skin anomalies, mild to severe intellectual disability (>95%), more severe nervous system anomalies (e.g., seizures 50%), more musculoskeletal abnormalities, severe vision impairments, severe feeding difficulties
Costello syndrome (CS)	*HRAS*, *NRAS*	HCM (60–70%), multifocal atrial tachycardia, short stature (>95%), more oncologic manifestations, mild to moderate intellectual disability (>90%), more nervous system anomalies (e.g., Chiari I malformation), more musculoskeletal abnormalities, severe feeding difficulties

Adapted from [4]; *A2ML1* has been excluded here, based on the discussion of this gene as a cause of NSSD [7]. JMML: juvenile myelomonocytic leukemia; HCM: hypertrophic cardiomyopathy.

**Table 2:** Descriptive information of the participants.

Variant	*n*	Mean Age (SD)	Age Range (Years)	% Men	Education Level * (Median)	Neurological Features **	Psychotropics
*PTPN11*	61	25.2 (12.5)	6−54	48%	4	8%	17%
*PTPN11/NSML*	3	16.7 (7.6)	10−28	67%	3	0%	33%
*SOS1*	14	25.7 (10.7)	8−48	36%	5	21%	43%
*KRAS*	7	22.7 (16.0)	12−61	57%	3	29%	29%
*LZTR1*	5	26.2 (17.2)	11−58	40%	4	0%	0%
*RAF1*	4	15.0 (3.4)	11−20	25%	3	25%	0%
*SHOC2*	2	24.5 (6.5)	18−31	50%	3.5	0%	0%
*CBL*	2	29.5 (16.5)	13−46	50%	4	100%	0%
*SOS2*	2	26.5 (8.5)	18−35	50%	4.5	0%	50%

* Education level 1 (<primary school) to level 7 (academic degree), according to the Dutch educational system [67], which is comparable to the UNESCO International Standard Classification of Education [68]; ** Neurological features: abnormalities of the central nervous system in the present or in the past (e.g., nystagmus, epilepsy, hydrocephalus).

**Table 3:** Descriptive classifications for the cognitive domains per variant group.

Variant		Domain/Variable													
		FSIQ	Speed	Att	Verb Memory			Vis Memory		Executive Functioning				Soc Cognition	
					Enc	Recall	Recog	Enc	Recall	Flex	WM	Inh	Plan	Emot	Ment
*PTPN11*	*n*	60	60	31	58	58	52	51	50	59	56	57	60	52	58
	Higher (%)	5	15	3	22	12	n.a.	10	14	10	4	13	14	2	0
	Expected (%)	45	68	87	59	76	90	78	82	75	96	74	65	60	66
	Lower (%)	50	17	10	19	12	10	12	4	16	0	14	22	39	35
*PTPN11/NSML*	*n*	3	3	3	3	3	3	2	2	3	3	2	3	2	3
	Higher (%)	0	0	0	0	0	n.a.	0	0	0	0	0	0	0	0
	Expected (%)	67	67	67	67	67	67	100	50	33	67	50	67	50	33
	Lower (%)	33	33	33	33	33	33	0	50	67	33	50	33	50	67
*SOS1*	*n*	14	13	7	13	14	13	13	14	13	13	12	13	13	14
	Higher (%)	22	8	14	0	7	n.a.	0	0	31	0	25	8	8	0
	Expected (%)	64	46	43	77	72	100	77	79	69	85	75	69	69	71
	Lower (%)	14	46	43	23	21	0	23	21	0	15	0	23	23	29
*KRAS*	*n*	7	7	6	5	6	5	3	4	6	7	5	6	6	6
	Higher (%)	0	14	17	0	0	n.a.	0	0	0	14	20	0	0	0
	Expected (%)	57	57	83	80	100	100	67	100	67	57	60	50	83	67
	Lower (%)	43	29	0	20	0	0	33	0	33	14	20	50	17	33
*LZTR1*	*n*	5	5	4	5	5	5	4	4	4	2	3	4	4	4
	Higher (%)	20	60	25	0	0	n.a.	0	0	0	0	0	25	0	25
	Expected (%)	40	40	75	40	80	80	50	75	100	100	100	75	50	50
	Lower (%)	40	0	0	60	20	20	50	50	0	0	0	0	5	25
*RAF1*	*n*	4	4	3	4	4	4	3	4	4	2	3	4	4	4
	Higher (%)	0	0	0	0	25	n.a.	0	0	0	0	0	25	50	25
	Expected (%)	25	25	67	100	50	100	67	100	100	100	100	100	50	50
	Lower (%)	75	75	33	0	25	0	33	0	0	0	0	0	0	50
*SHOC2*	*n*	2	2	1	2	2	2	1	1	2	1	2	2	2	2
	Higher (%)	0	0	0	0	50	n.a.	0	100	0	0	0	0	0	0
	Expected (%)	0	50	100	100	50	100	0	0	50	100	100	50	50	100
	Lower (%)	100	50	0	0	0	0	100	0	50	0	0	50	50	0
*CBL*	*n*	2	2	1	2	2	1	1	1	2	1	2	2	2	2
	Higher (%)	0	0	0	50	0	n.a.	100	100	0	0	50	50	0	0
	Expected (%)	50	100	100	50	100	100	0	0	0	100	50	50	50	50
	Lower (%)	50	0	0	0	0	0	0	0	100	0	0	0	50	50
*SOS2*	*n*	2	2	2	2	2	2	1	2	2	2	2	2	2	2
	Higher (%)	0	0	50	0	50	n.a.	100	50	0	50	50	50	0	0
	Expected (%)	50	100	50	50	50	100	0	0	100	50	50	50	100	100
	Lower (%)	50	0	0	50	0	0	0	50	0	0	0	0	0	0

FSIQ = full scale intelligence quotient; Att = attention; Verb Memory = verbal memory; Vis Memory = visual memory; Soc Cognition = social cognition; Enc = encoding; Recog = recognition; Flex = flexibility; WM = working memory; Inh = inhibition; Plan = planning; Emot = emotion recognition; Ment = mentalizing. n.a. = not applicable for this binary variable. Highlighted in dark grey are the variation group/cognitive construct combinations with significantly deviant performances; highlighted in light grey are the variation group/cognitive construct combinations that show a trend towards deviant performances.

**Table 4:** Descriptive classifications for the psychopathology measures per variant group.

Variant		Alexithymia	Internalizing	Externalizing	Thought Disorder
*PTPN11*	*n*	53	59	54	48
	Absent (%)	26	34	67	94
	Present (%)	74	66	33	6
*PTPN11/NSML*	*n*	2	3	3	2
	Absent (%)	0	33	67	50
	Present (%)	100	67	33	50
*SOS1*	*n*	12	14	14	14
	Absent (%)	8	36	86	93
	Present (%)	92	64	14	7
*KRAS*	*n*	4	4	4	4
	Absent (%)	50	75	100	100
	Present (%)	50	25	0	0
*LZTR1*	*n*	3	4	4	4
	Absent (%)	33	50	100	100
	Present (%)	67	50	0	0
*RAF1*	*n*	3	4	4	4
	Absent (%)	33	50	100	100
	Present (%)	67	50	0	0
*SHOC2*	*n*	2	2	2	2
	Absent (%)	0	100	100	100
	Present (%)	100	0	0	0
*CBL*	*n*	2	2	2	2
	Absent (%)	0	50	100	100
	Present (%)	100	50	0	0
*SOS2*	*n*	2	2	2	2
	Absent (%)	0	50	100	100
	Present (%)	100	50	0	0

Highlighted in dark grey are the variation group/cognitive construct combinations with significantly deviant performances; highlighted in light grey are the variation group/cognitive construct combinations that show a trend towards deviant performances.

## Data Availability

Data are available from the corresponding author upon reasonable request.

## Supplementary Materials

The following supporting information can be downloaded at: https://www.mdpi.com/article/10.3390/jcm11164735/s1, Table S1: Neuropsychological tests and questionnaires for each (cognitive) domain, Figure S1: Bootstrap distribution of point estimates (percentage of deviant scores) centered around the expected probability of deviant scores (either 32% or 16%), Figure S2: (a) Bootstrap distribution of point estimates (observed FSIQs) centered around the expected FSIQ of 100, (b) Bootstrap distribution of point estimates (difference in mean FSIQ between *PTPN11* and *SOS1*) centered around zero (no difference in means), Figure S3: Bootstrap distribution of point estimates (the difference in means between younger patients, aged ≤ 16, and older patients, aged > 16) centered around zero (no difference in means), and File S1: Explanation of the statistical methods.
