# Supplementary material for: Cognitive Phenotype and Psychopathology in Noonan Syndrome Spectrum Disorders through Various Ras/MAPK Pathway Associated Gene Variants
Source: J Clin Med. 2022 Aug 13;11(16):4735. doi: 10.3390/jcm11164735 (PMC9410383; doi:10.3390/jcm11164735)
Supplement: Supplementary file 1 [file jcm-11-04735-s001.zip › Figure S1.pdf]

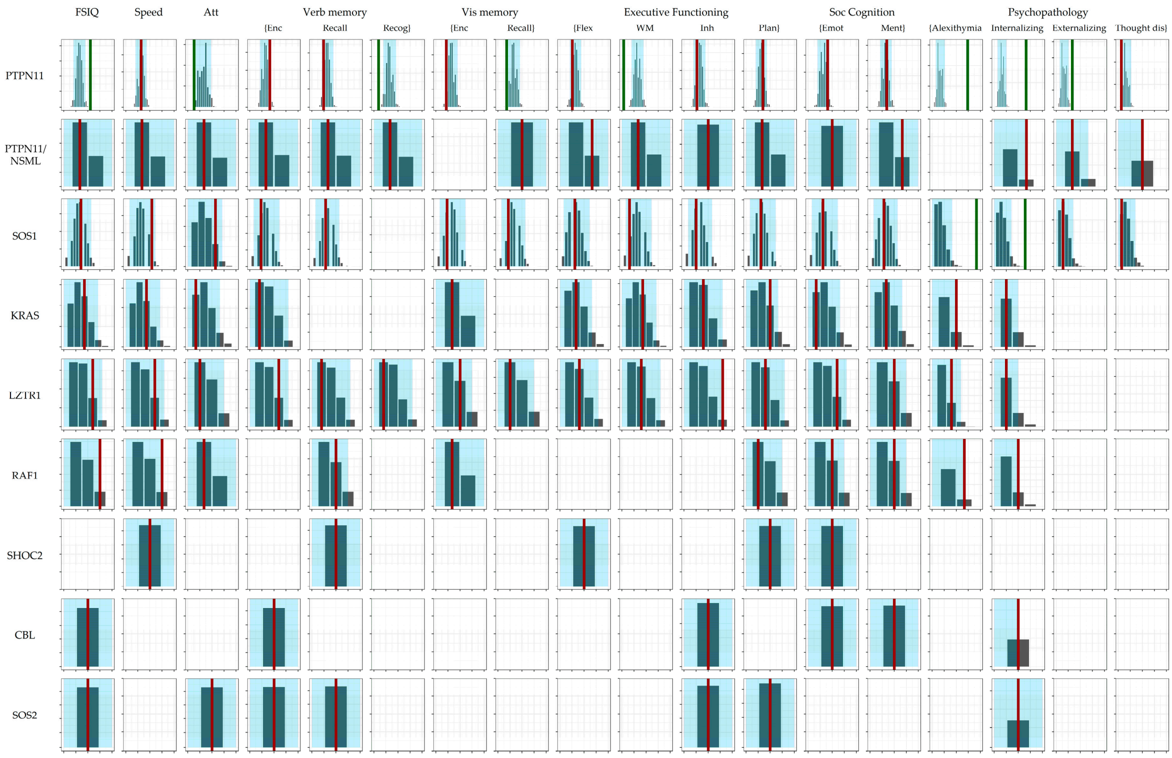

Figure S1. Bootstrap distribution of point estimates (percentage of deviant scores) centered around the expected probability of deviant scores (either 32% or 16%).
