# Supplementary material for: Cognitive Phenotype and Psychopathology in Noonan Syndrome Spectrum Disorders through Various Ras/MAPK Pathway Associated Gene Variants
Source: J Clin Med. 2022 Aug 13;11(16):4735. doi: 10.3390/jcm11164735 (PMC9410383; doi:10.3390/jcm11164735)
Supplement: Supplementary file 1 [file jcm-11-04735-s001.zip › File S1.pdf]

Supplementary File S1. Explanation of the statistical methods.

In this study, a structured clinical approach – taking into account multiple test outcomes, clinical observations and corrections for age and intelligence (see paragraph 2.3) – was used to classify individual test performances on 18 cognitive and psychological constructs of 100 patients with an NSSD as 'expected/normal' (-1 to +1 SD) or 'not-expected/deviant' (<-1SD and >+1SD). In a sufficiently large sample, this results in a normal distribution of sample means, in which 68% of the population has an expected score and 32% has a score that is 1 SD above or below this expectation. Using simulation-based methods, we tested whether the proportion of deviant (observed) scores in our sample was equivalent to the point estimate in the (simulated) population. For each variable/variant group combination, the observed values were resampled 5,000 times with replacement (bootstrap test) to simulate the population distribution. The distribution for each nonbinary variable was centered around the expected proportion of 32% deviant scores and the 95% confidence interval was calculated. Due to the clinical approach in combination with these analyses we were able to study cognitive phenotypes within variant groups, while controlling for moderating variables that might otherwise cloud the results.

Simulation-based methods were chosen instead of theory-based methods, because they provide a better understanding of the results by visualizing the sampling distributions and their corresponding confidence intervals. Especially in the variant groups with smaller sample sizes, these sampling distributions prevent under- and overinterpretation of raw scores. Simulation-based methods are less susceptible to violations of distributional assumptions. However, they do not provide a solution for the lack of information in small groups. Therefore, our results were further interpreted by inspecting raw scores. A rule of thumb (33.3% for groups with a sample size > 50, 66.6% for sample size >10, and 100% for smaller samples) was used to indicate deviations with potential clinical relevance.

After the initial analyses aimed at the 18 cognitive and psychological constructs, a series of exploratory simulation-based tests were performed on the FSIQs for all nine variant groups. FSIQs were bootstrapped 5,000 times for each group, and the resulting distributions were centered around an expected FSIQ of 100. For each group the 95% confidence interval was calculated. Furthermore, a confirmative simulation-based inferences test was performed to compare two of the variation groups (PTPN11 and SOS1) with respect to the FSIQ. To this end, FSIQs were resampled 5,000 times without replacement(permutation test) to simulate the population distribution of the difference of means between both groups, and the 95% confidence interval was calculated.

Finally, the whole sample was split into two age groups (≤16 years of age and >16 years of age) and confirmative simulation-based inferences tests were performed to compare performance of both age groups on all 18 variables. In these analyses, the original five classifications (ranging from low to high) were used. The observed values were permuted 5,000 times to simulate the population distribution of the difference in means between age groups, and the 95% confidence interval was calculated. For the binary variables the same method was applied, except a binary classification was used instead of the five-point classification.
