# Supplementary material for: Cognitive Phenotype and Psychopathology in Noonan Syndrome Spectrum Disorders through Various Ras/MAPK Pathway Associated Gene Variants
Source: J Clin Med. 2022 Aug 13;11(16):4735. doi: 10.3390/jcm11164735 (PMC9410383; doi:10.3390/jcm11164735)
Supplement: Supplementary file 1 [file jcm-11-04735-s001.zip › Table S1.pdf]

Supplementary Table S1. Neuropsychological tests and questionnaires for each (cognitive) domain.

Domain	Tests or questionnaires
Intelligence	WAIS-III, WAIS-IV, WISC-III, WISC-IV, WISC-V, SON-R 6-40*, RAKIT-2*, KAIT*
Speed of information processing	Processing Speed Index; WAIS-III, WAIS-IV, WISC-III, WISC-V, SON-R 6-40*<br>Baseline conditions; Stroop Color Word Test, (D-KEFS) Trail Making Test, D-KEFS Color Word Interference Test, d2 Cancellation Test, Bourdon-Vos Test*
Sustained attention	d2 Cancellation Test<br>Rapid Visual Information Processing; CANTAB<br>Bourdon-Vos Test*<br>Paced Auditory Serial Addition Task*
Executive functioning	
Inhibition	Interference condition; Stroop Color Word Test, D-KEFS Color Word Interference Test<br>Inhibition subtests; NEPSY-II*, IDS-2*
Cognitive flexibility	Flexibility conditions; (D-KEFS) Trail Making Test, D-KEFS Color Word Interference Test<br>Wisconsin Card Sorting Test<br>Intra-Extra Dimensional Set Shift; CANTAB<br>Flexibility subtests; BADS*, BADS-C*
Working memory	Working Memory Index; WAIS-III, WAIS-IV-NL, WISC-V-NL<br>Subtest Digit Span; WISC-III-NL*<br>Spatial Working Memory, Spatial Span; CANTAB*<br>Circle span task; NETOL*
Planning abilities	Tower of London<br>D-KEFS Tower Test<br>Test Stockings of Cambridge; CANTAB<br>Total score (or separate subtests*); BADS, BADS-C<br>Subtest Mazes; WISC-III, RAKIT-2*<br>Subtest Route Finding; NEPSY-II*
Episodic memory	
Verbal memory	Rey Auditory Verbal Learning Test for children and adults<br>Subtests Narrative Memory, Sentence Repetition, Word List Interference; NEPSY-II*<br>Subtests Eight Words Test, Story; NETOL*<br>Subtest Stories; IDS-2*<br>Subtest Stories; Rivermead Behavioural Memory Test – Third Edition*
Visual memory	Rey-Osterrieth Complex Figure Test<br>Location Learning Test – Revised<br>Paired Associates Learning; CANTAB*<br>Subtests Memory for Designs, Memory for Designs Delayed; NEPSY-II*<br>Subtest Visual Memory; NETOL*<br>Subtests Picture Description, Recognizing Figures, Recognizing Rotated Figures; IDS-2*<br>Visual Association Test (- Extended)*
Social cognition	
Emotion recognition	Emotion Recognition Task<br>Subtest Affect Recognition; NEPSY-II
Mentalizing abilities	Theory of Mind Test Revised<br>Subtest Theory of Mind; NEPSY-II<br>Faux-Pas Task<br>Strange Stories Test<br>Social Interpretation Test*

	ADOS(-2)*
General psychopathology	Screening questionnaires and Structured Clinical Interviews* of the DSM-IV; SCID-I and II<br>Shedler-Westen Assessment Procedure (SWAP)*<br>MMPI-2, MMPI-2-RF, MMPI-A*<br>NEO Personality Inventory-3 (NEO-PI-3)*<br>Screening of the Dimensional Assessment of Personality Pathology (DAPP)*<br>Five-Factor Personality Inventory (FFPI)*<br>Symptom Checklist-90 Revised (SCL-90-R)<br>Outcome Questionnaire (OQ-45)*<br>Spielberger State-trait Anxiety Inventory (ZBV)*<br>FEEL-E*, FEEL-KJ*<br>Scale for Interpersonal Behavior (SIB)<br>Inventory of Interpersonal Situations (IOA)*<br>Beck Depression Inventory-II (BDI-II-NL)<br>Hamilton Rating Scale for Depression (HDRS)*<br>Self-Rating Depression Scale (SDS)*<br>Children's Depression Inventory (CDI)*<br>Child Behavior Checklist (CBCL), Adult Behavior Checklist (ABCL)*<br>Strengths and Difficulties Questionnaire (SDQ)*
Alexithymia	Twenty-item Toronto Alexithymia Scale (TAS-20)<br>Bermond–Vorst Alexithymia Questionnaire (BVAQ)<br>Alexithymia Questionnaire for Children (AQC)*

Note. More details on the individual tests and their administration, scoring, and norms can be found elsewhere [69,70–72].

*Tests and questionnaires which were used only occasionally (less than 10 times).

WAIS-III = Wechsler Adult Intelligence Scale third edition; WAIS-IV-NL = Wechsler Adult Intelligence Scale fourth edition; WISC-III-NL= Wechsler Intelligence Scale for Children third edition; WISC-V-NL = Wechsler Intelligence Scale for Children fifth edition; SON-R 6-40 = Non-verbal intelligence test; RAKIT-2 = Revised Amsterdam Children's Intelligence Test; KAIT = Kaufman – Adolescent and Adult Intelligence Test; D-KEFS = Delis-Kaplan Executive Function System; CANTAB = Cambridge Neuropsychological Test Automated Battery; IDS-2 = Intelligence and Development Scales – 2nd Edition; BADS = Behavioural Assessment of the Dysexecutive Syndrome; BADS-C = Behavioural Assessment of the Dysexecutive Syndrome in Children; NETOL = Neuropsychological Test Series for Elderly People with Mild Intellectual Disability; ADOS = Autism Diagnostic Observation Schedule; MMPI = Minnesota Multiphasic Personality Inventories.
